# Exosome therapeutics for COVID‐19 and respiratory viruses

**DOI:** 10.1002/VIW.20200186

**Published:** 2021-01-31

**Authors:** Kristen D. Popowski, Phuong‐Uyen C. Dinh, Arianna George, Halle Lutz, Ke Cheng

**Affiliations:** ^1^ Department of Molecular Biomedical Sciences North Carolina State University Raleigh North Carolina USA; ^2^ Comparative Medicine Institute North Carolina State University Raleigh North Carolina USA; ^3^ Department of Molecular and Structural Biochemistry North Carolina State University Raleigh North Carolina USA; ^4^ Department of Biological Sciences North Carolina State University Raleigh North Carolina USA; ^5^ Joint Department of Biomedical Engineering University of North Carolina at Chapel Hill and North Carolina State University Raleigh/Chapel Hill North Carolina USA; ^6^ Division of Pharmacoengineering and Molecular Pharmaceutics University of North Carolina at Chapel Hill Chapel Hill North Carolina USA

**Keywords:** COVID‐19, exosomes, respiratory viruses, SARS‐CoV‐2

## Abstract

Respiratory viral diseases are a leading cause of mortality in humans. They have proven to drive pandemic risk due to their complex transmission factors and viral evolution. However, the slow production of effective antiviral drugs and vaccines allows for outbreaks of these diseases, emphasizing a critical need for refined antiviral therapeutics. The delivery of exosomes, a naturally secreted extracellular vesicle, yields therapeutic effects for a variety of diseases, including viral infection. Exosomes and viruses utilize similar endosomal sorting pathways and mechanisms, providing exosomes with the potential to serve as a therapeutic that can target, bind, and suppress cellular uptake of various viruses including the novel severe acute respiratory syndrome coronavirus 2. Here, we review the relationship between exosomes and respiratory viruses, describe potential exosome therapeutics for viral infections, and summarize progress toward clinical translation for lung‐derived exosome therapeutics.

## Graphical abstract

The delivery of exosomes, a naturally secreted extracellular vesicle, yields therapeutic effects for a variety of diseases, including viral infection. Exosomes and viruses utilize similar endosomal sorting pathways and mechanisms, providing exosomes with the potential to serve as a therapeutic that can target, bind, and suppress cellular uptake of various viruses including the novel SARS‐CoV‐2 coronavirus.

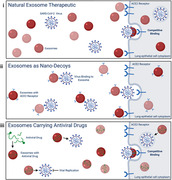

## 1 INTRODUCTION

Respiratory viral diseases are a leading cause of mortality worldwide, resulting in economic and disease burden. Respiratory viruses can spread through airborne transmission, notably through droplets and aerosols, as well as direct and indirect contact with hosts or objects.^1^ Transmissibility is complex and depends on many factors including environmental conditions, hygiene practices, and antiviral implications.^1^, ^2^ The complexity of respiratory viral diseases coupled with novel viral emergences makes maintaining and treating them a recurring issue. With exceptionally high transmissibility and the possibility for subsequent rebounding outbreaks, there is a critical race for effective antiviral drugs to cure current respiratory viral infections and to prevent future outbreaks.

Three common respiratory viruses including coronaviruses, influenza viruses, and enteroviruses have led to large‐scale outbreaks and pandemics, with the novel severe acute respiratory syndrome coronavirus 2 (SARS‐CoV‐2) causing the most recent pandemic. SARS‐CoV‐2 originally emerged from Wuhan, China in late 2019 and has since spread worldwide, causing the coronavirus disease of 2019 (COVID‐19) pandemic declared on March 11, 2020 by the World Health Organization.^3^ Currently, there are no therapeutic options that reliably and effectively cure COVID‐19. Ultimately, a vaccine against SARS‐CoV‐2 would be needed to offer protection and control the spread of the virus. However, with unprecedented pubic and private efforts, two vaccines are now approved by the FDA in a record‐breaking 11 months from the viral genome release. Though the vaccine protects the recipient from developing severe disease, there is no data thus far that shows the vaccine prevents continual transmission of the virus. Therapeutics have been developed for respiratory diseases like influenza viruses and enteroviruses, but do not provide a cure. Vaccines against influenza virus are notoriously hindered by antigenic drift, causing difficulty in developing a universal vaccine. Although annual vaccination is efficient and cost‐effective, it is highly dependent on the antigenic closeness of the circulating influenza virus.^4^ In addition, efficacy can be hindered by a compromised host immune response.^5^, ^6^, ^7^, ^8^ Development of an antiviral therapeutic that can evade a host immune response may provide an effective therapeutic for the immunocompromised. Antiviral drugs have also been developed for enteroviruses, but are challenged by the viruses’ diverse genomes and mutagenicities. With a focus on capsid inhibition, three potential targets ‐ pleconaril, BTA‐798, and V‐073 ‐ have undergone clinical trials in March 2002 and August 2010, and preclinical development in October 2009, respectively.^9^, ^10^ However, these targets lack activity in some enteroviral infections and are susceptible to drug resistance.^10^ Furthermore, many treatments for enteroviruses are supportive, rather than possessing antiviral properties. Approved treatments for enteroviruses only aim to control symptoms because there are currently no effective drugs or cure.^11^ Although recent clinical studies demonstrate a repression of viral spread, the need for an antiviral medication or treatments continues to be unmet.

There is an urgent need for strategies to contain and reduce viral spread. Exosomes offer a novel mode of delivery that not only deliver antiviral drugs, but also deliver their native cargo components. Extracellular vesicles, particularly exosomes, are naturally secreted by most cell types and are thought to play an important role in cell‐to‐cell communication. Exosomes function as a protective nano‐sized carrier of signaling payloads containing proteins, lipids, and microRNAs (miRNAs) that can cross biological barriers and utilize intracellular trafficking mechanisms for transport. They have an intrinsic ability to present specific surface proteins, such as CD47 and PD‐L1, which helps them protect their cargo and evade the immune system during circulation.^12^, ^13^ Exosomes offer an acellular approach to viral infection treatment by utilizing host machinery and molecular components to target and suppress viral replication. Therefore, exosomes may offer a novel therapeutic approach to contain viral spread.

## 2 EXOSOME AND VIRUS SIMILARITIES

Exosomes and enveloped viruses share similar features and purposes within a host. Containing biologically active material, both exosomes and viruses use a vesicular membrane to protect and deliver such material to either maintain tissue and microenvironmental homeostasis, or to preserve an infectious state.^14^ Although both are evolutionarily unrelated, exosomes and viruses can use endosomal sorting complexes required for transport (ESCRT) machinery to promote their biogenesis.^15^, ^16^ Both particles acquire their distinctive membranes through ESCRT‐dependent budding or shedding, but may contain different adaptor protein sites or late assembly domains that alter nanoparticle integration and recruit different components within the ESCRT pathway.^16^ Interestingly, several viruses are known to incorporate their viral material into extracellular vesicles as a disguising mechanism against host recognition and as protection against antibody neutralization.^17^, ^18^, ^19^ Together, our knowledge of exosomal and viral biogenesis can help elucidate targeting mechanisms for exceptional exosome therapeutics of viral infections.

### 2.1 Coronaviruses

Coronaviruses (CoVs) are a group of enveloped viruses with crown‐like spike protein complexes throughout the membrane surface. Under the family *Coronaviridae*, coronaviruses can infect both humans and animals with high pathogenicity ranging from mild disease, such as the common cold, to severe respiratory disease and death. Recent outbreaks of severe acute respiratory syndrome coronavirus (SARS‐CoV), Middle East respiratory syndrome‐related coronavirus (MERS‐CoV), SARS‐CoV‐2 have demonstrated the critical need for novel therapeutics that can reduce viral infection. Coronaviruses and exosomes are unique, naturally occurring nanoparticles, which share many similar features. Morphologically, coronaviruses are spherical, membrane‐enclosed virions with a diameter measuring 50‐200 nm, and exosomes are saucer‐shaped, lipid bilayer‐enclosed vesicles with a diameter of 30‐100 nm.^20^, ^21^ Both coronaviruses and exosomes can be transmitted through respiratory droplets and aerosols of approximately 5 µm in diameter, allowing upper and lower respiratory delivery to the lung.^22^ The unique proteins and receptors on coronaviruses and exosomes allow both types of particles to bind and be uptaken into recipient cells, such as lung epithelial cells. Once the recipient cells uptake either of these particles, coronavirus particles and exosomes work to modulate the cellular microenvironment through cargo exchange and release.^23^, ^24^ Both types of particles function as a carrier for their specific biomolecular cargo, such as DNA, RNA, and proteins. Coronaviruses, such as SARS‐CoV‐2, invade cells and hijack cellular machinery for viral replication and excretion of infectious progeny.^16^, ^25^ Exosomes transfer RNAs, including miRNAs, and proteins as a means of intercellular communication and paracrine signaling.^21^ Because coronaviruses and exosomes possess many similar features such as morphology, entry mechanisms, cell targeting ability, and aerosolization capability, exosomes present a promising new avenue for therapeutic development against SARS‐CoV‐2 with enhanced viral targeting and replication suppression.

### 2.2 Influenza viruses

Influenza viruses are single stranded negative‐sense RNA viruses under the *Orthomyxoviridae* family. Influenza viruses can be transmitted through aerosols or respiratory droplets, where they are uptaken into and replicated by the lung epithelium.^26^ The virus is classified into three serotypes (A, B, and C), with influenza A virus (IAV) causing global pandemics such as the Spanish Flu in 1918 and the swine flu in 2009. IAV is divided into hemagglutinin and neuraminidase subtypes, altering pathogenesis and antigenicity.^27^ Previous studies have demonstrated that host miRNAs defend against influenza viral infection, leading to the inhibition of replication and reduction of pathogenicity.^28^, ^29^, ^30^ Similarly, exosomes utilize miRNAs in host defense. During influenza virus infection, exosomes have been shown to transport viral proteins to neighboring cells, which, in turn, activate a host immune response against the virus.^31^ Particularly, cells infected with influenza virus demonstrate miRNA upregulation and packaging into exosomes, which were secreted and uptaken by neighboring cells to induce interferon expression.^32^ This interconnection of exosomal and viral biogenesis pathway utilization and cargo packaging has expanded research to exploit such interconnections, to help combat viral infection.^33^

### 2.3 Enteroviruses

Enteroviruses are single stranded RNA‐containing viruses with capsids approximately 15‐30 nm in diameter. Enteroviruses typically contain simple message sense genomes and express the ability to recombine with other enteroviruses. The genus *Enterovirus* infects higher vertebrates, including humans, through contact or fecal‐oral routes and has been known to cause a variety of diseases that range in severity.^11^ Oftentimes, enteroviruses cause short‐term diseases, such as the common cold; however, in other cases, they may result in damage to the central nervous system, encephalitis, myocarditis, poliomyelitis, acute heart failure, and sepsis.^11^, ^34^ Recent findings have demonstrated the integration of exosomal and viral mechanisms in enterovirus 71 (EV‐71) specifically, where exosomes from virus‐infected cells facilitate in viral replication through miR‐30a transfer and virion cloaking.^35^, ^36^ Additionally, miR‐155 has been found to inhibit EV‐71 infection by targeting phosphatidylinositol clathrin assembly protein (PICALM) in recipient cells.^37^ These mechanistic interactions indicate that exosomes are a promising drug delivery vesicle for enterovirus therapeutics.

## 3 EXOSOME THERAPEUTIC STRATEGIES

Various nanoparticle drug delivery systems have been utilized as clinical therapeutics including micelles, gold nanoparticles, and liposomes. Currently, liposomes are the leading candidates for COVID‐19 miRNA vaccine production.^38^, ^39^ However, such systems lack additional therapeutic components that may outperform traditional systems and overcome the biological barriers involved in drug delivery.^40^, ^41^, ^42^ Using several optimized strategies, exosomes can serve as a therapeutic platform for the treatment of many diseases that can overcome such obstacles. Exosomes have clinical applications as cell‐free alternatives for disease treatment and tissue regeneration by delivering therapeutic cargo components while circumventing immune rejection and cellular toxicity.^43^ Stem‐cell‐derived exosomes are particularly advantageous, harnessing the anti‐inflammatory and regenerative abilities of their parent cells.^44^ Coupled with derivation from native organ types, exosomal treatment can be engineered for respiratory viral diseases such as SARS‐CoV‐2.

**FIGURE 1:**
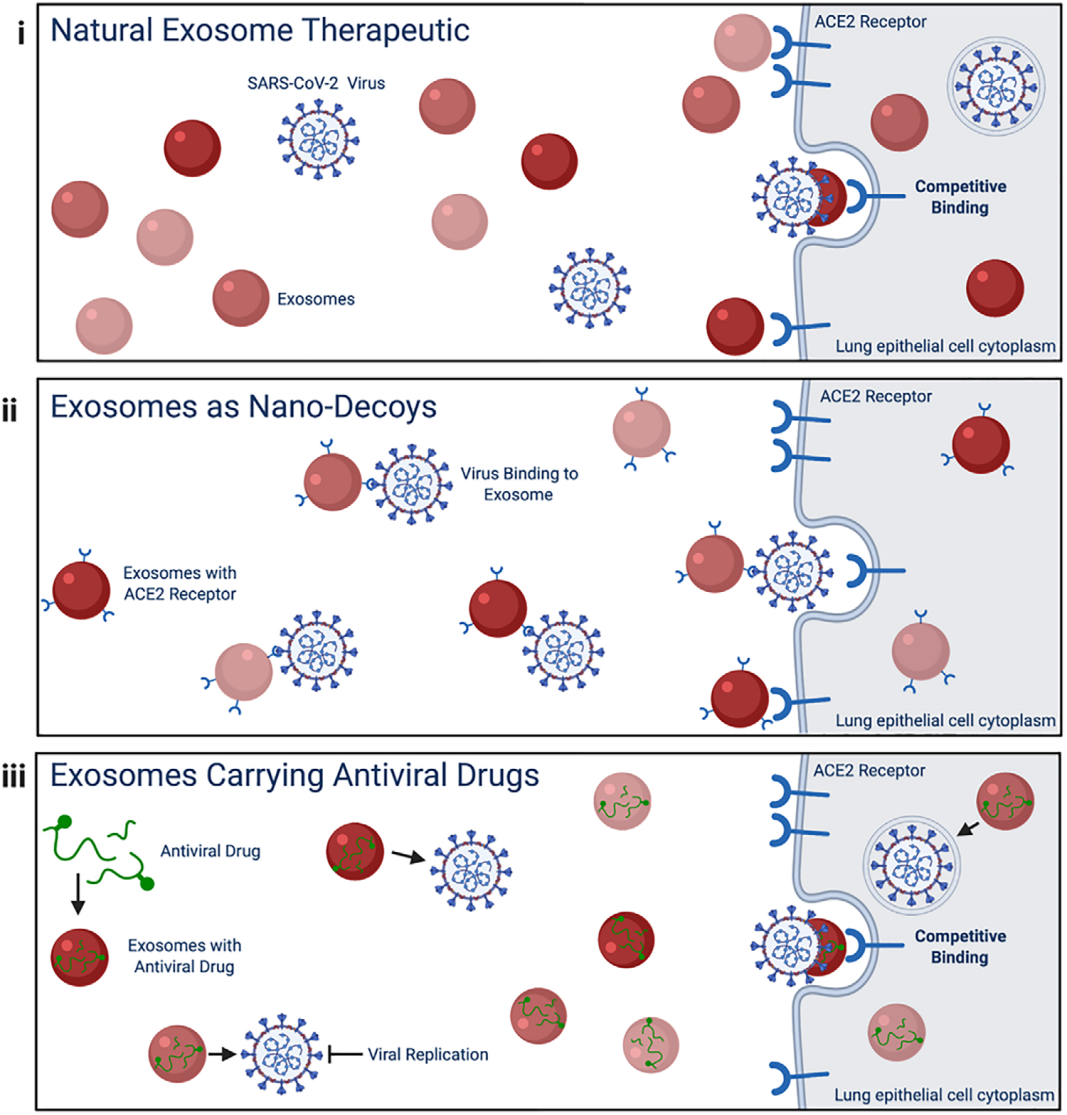
Therapeutic potential of natural and engineered exosomes for SARS‐CoV‐2. Proposed therapeutic mode of action of (i) natural exosomes, (ii) engineered ACE2 receptor presenting exosomes as a binding nano‐decoy, and (iii) engineered exosomes containing antiviral drugs for potential therapeutic approaches against the SARS‐CoV‐2 virus.

Lung‐derived exosomes have shown superior therapeutic benefits in the lungs over their exogenous counterparts, such as mesenchymal stem cell exosomes, offering a refined therapy.^45^ Delivery of such exosomes to treat respiratory viral infections would provide additional therapeutic benefits to counteract the systemic cytokine storm and multi‐organ damage as seen in COVID‐19 patients (Figure 1i).^46^ Another potential strategy includes utilizing exosomes as “nanodecoys” to bind the viruses and prevent viral uptake into host cells. Comprising of membrane proteins and receptors from the parent cell, exosomes would naturally trap and detain the virus that would otherwise infect the host cell.^47^ Utilization of exosomes as cell‐mimicking “nanodecoys” would slow viral infection by binding and tagging the virus for immune cell elimination. Previous studies have demonstrated that cell‐membrane‐mimicking nanostructures successfully entrap pathogens and are not prone to membrane fusion with host cells, allowing the host immune system to clear the entrapped pathogens before infection.^48^, ^49^, ^50^ Additionally, exosomes have been shown to directly trap membrane‐acting virulence factors, such as pore‐forming toxins, to prevent viral uptake by host cells.^51^ Such entrapment strategies can allow for neutralization of host inflammation following viral infection, emphasizing the mechanistic similarities between exosomes and viruses and the potential to optimize exosome “nanodecoys.”^52^ Recent studies have also shown that type II alveolar epithelial cells in the lungs and goblet secretory cells in the nasal mucosa are specific cellular targets of the SARS‐CoV‐2 virus via binding of the viral spike (S‐) protein to angiotensin‐converting enzyme 2 (ACE2) on the cell surface membrane for viral entry.^53^ Therefore, lung‐derived exosomes that present ACE2 on their surfaces may be superior decoys (Figure 1ii). These ACE2 containing lung exosomes are naturally occurring and have the specific membrane‐bound ligands and receptors necessary to effectively bind SARS‐CoV‐2 and prevent cellular entry. Aside from naturally occurring exosomes, engineered exosomes as drug carriers to deliver specific payloads is another therapeutic strategy. Antiviral drugs can be loaded into exosomes, which act as carriers, to provide enhanced delivery to targeted organs such as the lungs, while minimizing off‐target effects (Figure 1iii). Specifically, in terms of the COVID‐19, antiviral‐loaded exosomes can be delivered intranasally directly to the nasal mucosa and the lungs, the two sites most frequently exposed to the coronavirus. Previous synthetic drug delivery systems are limited by their cytotoxicity, immunogenicity, and/or delivery inefficiency.^54^ Encapsulating drugs into exosomes evades such limitations and may provide superior recipient cell targeting.^55^ Coupled with their innate anti‐inflammatory effects, exosomes carrying SARS‐CoV‐2 antiviral therapeutics may provide superior suppression of viral replication and its subsequent diseases such as cytokine storm syndrome and acute respiratory distress syndrome (ARDS) often observed in COVID‐19 patients.

Exosomes can also be optimized for influenza viruses and enteroviruses, by coupling antiviral drugs with native exosomal therapeutic components to combat successive lung inflammation. Utilizing exosomes from lung or lung‐secretion may provide a specialized acellular therapeutic with native antiviral properties that can help control viral replication and host inflammation.

The main site for influenza infection is the respiratory mucosa, infecting both the upper and lower airways. Exosomes have been found in respiratory secretions, such as saliva and bronchoalveolar fluid, and have known antiviral effects.^56^, ^57^, ^58^, ^59^, ^60^, ^61^, ^62^, ^63^, ^64^ Recently, studies have found that exosomes derived from human bronchoalveolar fluid and tracheobronchial epithelial (HTBE) cell culture secretions have antiviral properties, suggesting that exosomes isolated from these sources may provide a refined therapy for influenza virus.^57^, ^65^ In addition, cargo within exosomes may play a role in the host innate immune response following influenza exposure. Exosomes from HTBE cells showed association of α‐2,6‐linked sialic acid with epithelial mucins and subsequent neutralization effects on human influenza virus.^65^ Exosomes from the bronchoalveolar lavage fluid of influenza‐infected mice contain miRNAs that may also mediate the antiviral and inflammatory response.^66^ Altogether, exosomes from such sources may better control viral replication and lung inflammation. Loading anti‐influenza drugs into these exosomes could provide a specialized therapeutic with more efficient patient outcomes than exosomes or antiviral drugs alone.

Exosomes may also be utilized as potential therapeutics for enteroviral diseases as antiviral drug carriers. Due to the nature and genetic diversity of this genus, enteroviruses pose an ongoing threat for the potential outbreaks and epidemics.^34^ Some clinical studies show promise by identifying mechanisms of dismantling enterovirus replication.^9^, ^34^ These findings report a collection of molecules that express the ability to bind to enterovirus capsids, preventing the attachment of the viral proteins necessary for attachment and uncoating.^34^, ^67^ Capsid‐binding agents, such as pleconaril, pirodavir, and vapendavir, have been developed to combat enteroviruses, but show limited antiviral activity, poor bioavailability, and intrinsic resistance.^9^, ^34^, ^68^ Additionally, in a double‐blinded, placebo‐controlled trial of pleconaril, several factors including small study size and low serum concentration of pleconaril made it difficult to assess its therapeutic potential.^9^, ^68^ Loading these capsid‐binding agents into exosomes may enhance their antiviral activities and potency, since exosomes utilize enteroviral mechanisms.

Overall, exosomes are a customizable platform for drug delivery and immune regulation. By deriving exosomes from sources optimized for respiratory viral infections, natural exosomes, exosomes as nano‐decoys, and exosomes carrying antiviral drugs all pose as potential therapeutic strategies.

## 4 CLINICAL TRANSLATION PATHWAY

**FIGURE 2:**
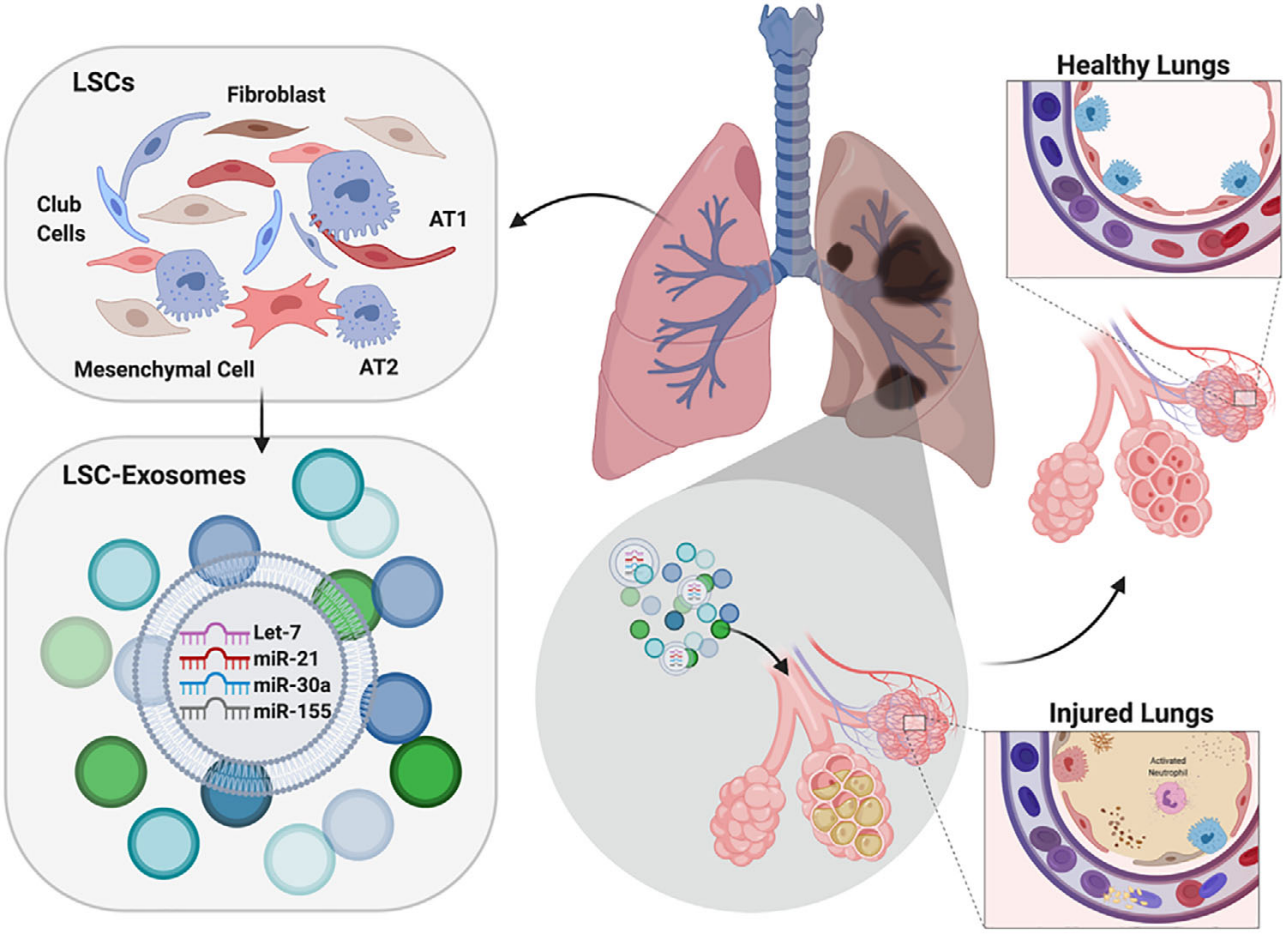
Proposed therapeutic mechanisms of inhaled LSC‐Exosomes. Proposed therapeutic mechanism of inhaled LSC‐exosomes treatment in injured lungs from respiratory viruses Abbreviations: AT1, alveolar type I pneumocyte; AT2, alveolar type II pneumocyte; LSC‐exosomes, lung spheroid cell‐exosomes; LSCs, lung spheroid cells.

The lungs have the natural ability to undergo facultative regeneration due to their resident stem and progenitor cell populations. Utilizing the lungs’ regenerative potential, therapeutic lung spheroid cells (LSCs) can be generated from adult lung cells.^69^, ^70^, ^71^ LSCs are currently being investigated in a first‐in‐human Phase 1 clinical trial of intrinsic lung‐derived cells for treating lung disease (NCT04262167). Additionally, it's been shown that not only LSCs, but also LSC secretome and exosomes are capable of tissue regeneration in multiple lung injury models, including bleomycin‐induced pulmonary fibrosis and crystalline silica‐induced silicosis.^46^, ^71^, ^72^, ^73^ In an endeavor to optimize the advantages of LSCs and exosomes, we propose the potential therapeutic opportunity presented by human LSC‐exosome inhalation in patients with lung injury such as COVID‐19‐associated ARDS by utilizing both the targeting ability of ACE2 from lung cell membrane as well as anti‐inflammatory miRNAs (**Figure** **2**).

## 5 CONCLUSION

Altogether, it is clear that exosomes provide a diverse array of strategies to combat respiratory viruses, including SARS‐CoV‐2, to address the current COVID‐19 global pandemic. Here we presented: (a) natural exosome, (b) ACE2 presenting exosomes as a nano‐decoy, and (c) antiviral loaded exosomes as potential acellular therapeutic approaches against the SARS‐CoV‐2 virus, influenza viruses, and enteroviruses. Although evolutionarily independent, exosomes and respiratory viruses such as coronaviruses, influenza viruses, and enteroviruses may utilize sorting complexes and cellular mechanisms that help maintain host homeostasis or viral replication. Developing exosome therapeutics as natural exosomes, nano‐decoys, or antiviral loaded exosomes provides options for treatment optimization that can target, bind, and suppress cellular uptake of various viruses. Due to their natural role in intercellular communication and inherent immunogenicity and cytotoxicity, exosomes pose great potential as a natural therapeutic as well as a novel drug delivery vehicle.

## CONFLICT OF INTEREST

Ke Cheng is a co‐founder and equity holder of BreStem Therapeutics and Xollent Biotechnology. BreStem and Xollent provided no funding to this study. The remaining authors declare no conflict of interest.
